# Risk factors of social anxiety in patients with essential tremor

**DOI:** 10.3389/fpsyt.2023.1051290

**Published:** 2023-02-06

**Authors:** Lijun Hou, Xiaoguang Lei

**Affiliations:** ^1^Department of Neurology, Qujing Second People’s Hospital, Qujing, China; ^2^Department of Neurology, The First Affiliated Hospital of Kunming Medical University, Kunming, China

**Keywords:** essential tremor (ET), social anxiety (SA), non-motor symptoms (NMS), social anxiety in essential tremor (ET-SA), Liebowitz Social Anxiety Scale (LSAS), Fahn-Tolosa-Marin Tremor Rating Scale (FTMTRS)

## Abstract

**Objective:**

To study the risk factors of social anxiety (SA) in essential tremor (ET) patients.

**Methods:**

Motor, cognition, and SA were evaluated using the Fahn-Tolosa-Marin Tremor Rating Scale (FTMTRS), Mini-Mental State Examination (MMSE), and Liebowitz Social Anxiety Scale (LSAS) for each subject. The potential risk factors of SA in ET were analyzed using univariate analysis.

**Results:**

A total of 80 ET patients and 85 healthy controls completed the evaluation. The LSAS evaluation showed that the prevalence of SA in the ET group was 48.8%, higher than that in controls (12.9%, *P* < 0.001). Female (*OR* = 4.959, *P* = 0.014), younger age (*OR* = 4.172, *P* = 0.037), and head tremor (*OR* = 4.707, *P* = 0.025) were risk factors of SA among ET patients.

**Conclusion:**

SA is prevalent in patients with ET. Risk factors, such as female sex, age, and head tremor, should be considered for the prevention and intervention of SA in ET patients.

## 1. Introduction

Essential tremor (ET) is the most common movement disorder clinically manifested by postural and kinetic tremors involving the hands, head, and other parts of the body (1). The prevalence of ET in the population is about 0.9% and increases with age, with a prevalence of about 4.6% in the elderly population over 65 years of age (2). ET, characterized by postural tremor, is traditionally considered a pure tremor disease. Recently, the non-motor symptoms of ET, including cognitive deficits, sleep problems, and hearing problems, have received more awareness (3–5). It is more considered a complex disease influenced by multiple genetic and environmental factors (6, 7), with motor and non-motor symptoms (8). The combination of these non-motor symptoms enormously affects the quality of life of ET patients. Recognition and management of these non-motor symptoms may improve the quality of life in ET patients.

According to DSM-IV diagnostic criteria (9), a diagnosis of social phobia may be excluded by the presence of ET. This finding may explain why early ET with social anxiety (SA) is often overlooked. However, several subsequent studies found that the symptoms of SA are more common in patients with ET than in the control group (10–13) which shows that SA can be a complication of ET.

Metzer (14) first observed that several ET patients reported severe embarrassment and decreased quality of life in the social field, due to uncontrollable tremors (15). Many patients with ET may commonly exhibit harm avoidance, specifically in the domains of pessimistic worrying and ruminating on humiliating and embarrassing situations (16). The avoidance of social contact as a defense strategy can lead to reduced activity and lower subjectivity in social participation, severe anxiety, depression, and suicide (17). Therefore, early screening and identifying of social anxiety in essential tremor (ET-SA) patients and timely intervention to the patients can improve the quality of life and save social and medical resources (10, 11, 13).

Identifying patients with risk factors and then giving them adequate attention will facilitate earlier detection and intervention for social-psychological problems, including SA. So far, there were researches reported that age and female may be the risk factors of ET-SA, but the features of tremor (like severity, distribution and so on) which are more relevant to the ET manifestation, are still not the warning flags to clinicians. In this study, we will try to reveal more risk factors, especially in features of tremor.

## 2. Materials and methods

### 2.1. Subjects

A total of 88 ET patients were enrolled from the inpatient and outpatient departments of Qujing Second People’s Hospital (in the northeastern part of Yunnan) in about 1.5 year from July 2019 to January 2021. Patients who met the diagnostic criteria for ET according to the Chinese guidelines (1), which is translated from criteria for the diagnosis of essential tremor (18), were included. The inclusion criteria were as follows: (1) obvious and persistent postural and/or action tremors in both hands and forearms; (2) no other neurological signs (except cogwheel phenomenon and Froment sign); (3) may have head tremor but not accompanied by dystonia. (4) course of the disease exceeding 3 years. The exclusion criteria in this study were as follows: (1) the presence of significant lesions in the brain on MRI which may cause tremors and mood problems; (2) sudden onset or rapid progression of the disease; (3) unilateral limb tremor; (4) tremor accompanied by rigidity and bradykinesia. Age >18 years; and (5) patients with past history of psychosis or have obvious symptoms like hallucination, delusion, depression (but not anxiety). 100 healthy controls with no brain disorders were enrolled in the control group. We collected basic characteristics, including age, sex, education, living environment, and family history, and evaluated motor, cognition, and social anxiety based on scales. We provided the subjects or guardians with adequate information and obtained written consent.

### 2.2. Scales

Three scoring scales were used for evaluation: Fahn-Tolosa-Marin Tremor Rating Scale (FTMTRS), with a maximum of 100 points, was used to evaluate the tremor location, operation, and ability of daily living, with a total of 21 items; Mini-Mental State Examination (MMSE), with a maximum of 30 points, was used to evaluate cognitive function, with 11 items. The cognitive function decline standard was illiteracy <17, primary school <20, junior high school, and above <24 (19). Liebowitz Social Anxiety Scale (LSAS) was used to evaluate social anxiety, including social situations, operating social situations, fear, anxiety, and avoidance. The evaluation time range was the situation in the last 3 months during the survey. The subjects received 0–3 points according to the severity of 4 grades (none, mild, moderate, and severe), and the total score “greater than 38” was set in this study to meet the “excessive” criteria in DSM-5 (20).

### 2.3. Statistical analysis

SPSS17.0 statistical software was used for data processing and analysis. The Chi-square test was used to test the composition ratio of the two groups of counting data. The measurement data of normal distribution were expressed as mean ± standard deviation ($\bar{X}$ ± *s*), in which the *t/t′* test of two independent samples was adopted. The measurement data with non-normal distribution were represented by median and quartile [*M (QR)*], and the Mann–Whitney *U* test was used. Logistic regression analysis with the entry method was used to screen for independent variables. The step probability p entry and deletion criteria were 0.05 and 0.10, respectively. *P* < 0.05 was considered statistically significant.

## 3. Results

### 3.1. Higher prevalence of ET-SA

A total of 8 ET patients and 15 controls failed to complete study because time inconvenience. Finally, 80 ET patients and 85 controls completed the evaluations. There was no statistical significance between the general conditions for both groups (*P* > 0.05, Table 1). The scores of social occasions and operational social scene were higher in ET group than that in control group (both *P* < 0.001). The prevalence of social anxiety in ET patients (48.8%) was significantly higher (*P* < 0.001) than that in the control group (12.9%, Table 2). There was no significant difference in the total tremor score and ET duration between ET patients with and without SA and ET patients (*P* = 0.365, Table 3).

**TABLE 1:** Demographic characteristics of essential tremor (ET) patients and controls.

Groups	Inpatient or outpatient	Family history	Sex male: female	Age (years)	Area urban: rural	Education (year)	MMSE
Control	0/85	0	49:36	49 (18)	50:35	7 (7.5)	26 (5)
ET	55/25	27	36:44	52 (10)	37:43	6 (5)	26 (7)
Test statistic	–	–	χ^2^ = 2.639	*Z* = −1.512	χ^2^ = 2.614	*Z* = −0.992	*Z* = −0.294
*P*	–	–	0.104	0.131	0.120	0.321	0.769

ET patients and controls were not statistically different in demographic characteristics (*P* > 0.05).

**TABLE 2:** Liebowitz Social Anxiety Scale (LSAS) scores for essential tremor (ET) and control groups.

Group	Score on social occasions	Score of operational social scene	Number (ratio) of subjects with social anxiety
Control	23.09 ± 11.35	30.32 ± 13.08	11 (12.9%)
ET	34.74 ± 15.33	39.08 ± 15.18	39 (48.8%)
Test statistic	*t′* = −5.52	*Z* = −3.792	χ^2^ = 25.021
*P*	<0.001*	<0.001*	<0.001*

There was no significant difference in LSAS total score and subgroup scores between ET and control groups (*P < 0.05).

**TABLE 3:** Single-variable analysis of risk factors for essential tremor patients with social anxiety (ET-SA) (General).

Groups	Sex male: female	Age (years)	Education (year)	Area urban: rural	Hereditary yes: no	ET course (year)	FTMTRS	MMSE decline: normal
ET-non-SA	28:13	38 (18)	6 (7)	20:21	12:29	7.1 (4.0)	30.94 ± 17.61	14/27
ET-SA	6:33	35 (19)	7 (8)	17:22	15:24	7.1 (4.9)	34.61 ± 18.27	4/35
Test statistic	χ^2^ = 22.895	*Z* = −1.881	*Z* = −1.212	χ^2^ = 0.661	χ^2^ = 0.756	*Z* = −0.338	*t* = −2.559	χ^2^ = 6.542
*P*	<0.001^#^	0.060^#^	0.226	0.217	0.480	0.735	0.365	0.015^#^

Two factors (sex, age) were selected and included in the SPSS analysis. Collinearity between age and cognitive function was found; the latter was not included in the equation (^#^*P* < 0.1).

### 3.2. Risk factors of ET-SA

In the univariate analysis, four factors, including sex, age, head tremor, and cognitive function, were significantly associated with ET-SA and included in the logistic regression equation as independent variables (Tables 3, 4) to evaluate the impact on the prevalence of the dependent variable ET-SA. We found collinearity between age and cognitive function (Variance inflation factor VIF: 9.750, condition index: 11.952). Therefore, we excluded cognitive function and included the remaining three independent variables in the equation. The final logistic model was statistically significant, χ*^2^* = 40.713, *P* < 0.01; the model could correctly classify 90.0% of the research subjects with a sensitivity of 92.3%. Of the three independent variables included in the model, age, sex, and head tremor were statistically significant. The risk of SA in female ET patients was 4.959 times higher than in males (*P* < 0.05). The risk of SA in young and middle-aged ET patients was 4.172 times higher than in older adults (*P* < 0.05). The risk of SA in ET patients with head tremor was 4.707 times higher than in ET patients without head tremor (*P* < 0.05, Table 5).

**TABLE 4:** Single-variable analysis of risk factors for essential tremor patients with social anxiety (ET-SA) (Tremor position).

Groups	Face	Sound	Tongue	Head	Upper limb	Trunk	Lower limbs
ET-non-SA	8	14	9	5	35	1	1
ET-SA	5	9	13	27	35	2	4
χ^2^	0.658	1.196	1.299	27.02	0.350	4.000	2.085
*P*	0.417	0.274	0.254	<0.001^#^	0.554	0.527	0.149

Among ET patients, only head tremor was associated with SA (^#^*P* < 0.1), and tremors in the rest of the body were not associated with SA (*P* > 0.1).

**TABLE 5:** Multivariable analysis of disease risk factors for essential tremor patients with social anxiety (ET-SA).

						(95% CI)	
***X***	***B***	***Sb***	**Wald χ^2^**	***P***	***OR***	**Lower**	**Upper**
Sex (female vs. male)	1.601	0.652	6.039	0.014*	4.959	1.383	17.781
Age (<60 vs. ≥60)	1.428	0.685	4.343	0.037*	4.172	1.089	15.989
Head tremor (yes vs. no)	1.549	0.692	5.012	0.025*	4.707	1.213	18.265

Three independent variables (sex, age, head tremor) included in the multivariate analysis were all statistically significant.

*P < 0.05.

## 4. Discussion

The lifetime prevalence of SA has been reported in various studies, ranging between 3 and 13% (21), while we found that nearly half (48.8%) of ET patients had the symptoms of social anxiety. This result was consistent with previous reports. A survey in Latvia found that the prevalence of SA in the ET group was higher than that in the control group, and the degree of tremor moderately correlated with the degree of SA (12). Ozel-Kizil et al. (11) found that 30% of ET patients suffer from varying degrees of SA (12). They showed that the severity of SA in ET patients negatively correlated with age, positively correlated with a social disability, and did not correlate with the severity and course of motor disorders (11). The above studies show that the prevalence of SA is higher in ET patients than in controls, which supports our study results. SA patients tend to avoid self-exposure. Therefore, those community-based studies may show a relatively lower prevalence of SA. Among ET patients, the ratio of inpatients to outpatients suffering from SA was 22:55 vs. 17:25, (*P* = 0.02), possibly because inpatients are exposed to more strangers, which increases their exposure to tremor, and therefore prefer to complete their consultations in the outpatient setting.

Not all ET patients experience SA. Our study showed that age, sex, and head tremor were the risk factors for ET-SA. Some studies have measured ET patients using the LSAS scale and found that the degree of ET tremor was positively related to the degree of SA, i.e., patients with higher tremor scores were at higher risk of developing SA (13, 22). However, it is also believed that studies have concluded that there is no correlation between the two (10). A Turkish study of risk factors in patients with Parkinson’s disease-SA found that masculinity and postural instability were risk factors for the development of SA (23). In contrast, in a Saudi Arabian student survey (21), the prevalence of SA was almost 51% among the 5,896 Saudi medical students who participated in the study. Younger age and females had a higher risk of developing SA (*OR* = 1.09 and 1.13).

Our study showed female ET patients are more likely to have SA. The mechanism may involve the effects of steroid hormones on neural excitability (24). Compared with males, female patients pay more attention to their external image and prefer to avoid social contact and its related embarrassment (25, 26). However, a Turkey study that show that males with Parkinson’s disease are more likely to suffer from SA (23). The inconsistency may be related to the type of primary disease and the degree of participation of men/women in social life in different countries, but further research is needed to confirm.

Our study also suggests that the risk of SA among young and middle-aged ET patients is higher than that in older ET patients. This probably because young ET patients pay more attention to external images (27). There were also histopathological studies revealed that ET may show slow degenerative changes in the whole brain (28, 29) and those degenerative cerebellum (30), cerebellothalamocortical pathway, limbic system and prefrontal cortex are all involved in controlling non-motor functions (31–33). However, research suggested that brain degeneration in ET patients leads to a decline in frontal lobe function (34) and cognitive impairment (MMSE declined, also showed in our study), which may leads to a more “calm” mentality and a decrease in external image attention. Besides, maturity with accumulated life experience and increased knowledge, may also contribute to a lower prevalence of SA in older ET patients than in young and middle-aged ET patients. Thus, the final effect of age is supposed to be the combination of all different factors.

Some studies have measured ET patients using the LSAS scale and found that the degree of ET tremor was positively related to the degree of SA, i.e., patients with higher tremor scores were at higher risk of developing SAD (12, 13, 22). However, there were also studies concluded that there is no correlation between the two (10). Our study showed no significant difference in the total FTMTRS score and the ET duration between ET patients with and without SA. This may because the phenotype heterogeneity and relatively small sample in this study. However, we found that the head tremor score correlated to the disease in ET-SAD patients, which is rarely reported. This finding links the motor symptoms with the non-motor symptoms of ET. Head tremor is the behavior that most easily attracts attention in social interactions, which can weaken the patient’s overall mental outlook and produce a lot of negative emotions (25). Head tremor is also related to the VI-VII lobules of the cerebellar vermis (limbic cerebellum), amygdala and hippocampus (27), which are involved in abnormal emotional processing, especially SAD (27).

According to our results, physicians can focus on screening female, young and head tremor patients to early reduce the physical and psychological suffering of patients. Early intervention or ET-SA treatment, including active thinking cessation, systemic desensitization, recognition of non-ideal thinking, other cognitive behavioral therapies (16). Treatment of SA has been suggested to be helpful for social anxiety secondary to ET and a variety of other conditions. Among ET patients, tremor exacerbations related to anxiety and stress may respond to treatment with β-blockers and benzodiazepines, both of which are also treatments for SA (22).

This study has some limitations. The small sample size may cause missing possible risk factors in the study. The patient sample from our hospital may introduce some common biases, for example, the patients may be the ones with more pronounced tremor and more impaired quality of life, compared with those patients who didn’t come to hospital. This study is based on hospital patients and may have biases that affect the severity of social anxiety (stress and attention from researcher and hospital environment may exacerbate tremor). We did not assess “quality of life” and “disability” in this study which may provide more complete image of impact of SA. Long-term follow-up is needed to reveal the nature course of ET-SA and confirm the effect of early intervention. This is a single-center cross-sectional study based on a local population in northeastern Yunnan Province, China. The need to combine the present results with those of other similar studies due to ethnic and cultural variability and the heterogeneous nature of ET.

In conclusion, ET-SA may be not only a neurodegenerative process but also a conditioned emotional response, with connection between motor and non-motor symptoms. This study identified age, sex, and tremor distribution as risk factors of ET-SA. Head tremor is given insufficient attention. ET-SA can be early recognized by using risk factors.

## Data availability statement

The original contributions presented in this study are included in this article/supplementary material, further inquiries can be directed to the corresponding author.

## Ethics statement

Ethical review and approval was not required for the study on human participants in accordance with the local legislation and institutional requirements. The patients/participants provided their written informed consent to participate in this study.

## Author contributions

LH analyzed and interpreted the data and drafted the manuscript. Both authors designed the study, acquire the data, revised the manuscript for important intellectual content, read, and approved the final manuscript.
